# Soil Lead and Children’s Blood Lead Disparities in Pre- and Post-Hurricane Katrina New Orleans (USA)

**DOI:** 10.3390/ijerph14040407

**Published:** 2017-04-12

**Authors:** Howard W. Mielke, Christopher R. Gonzales, Eric T. Powell

**Affiliations:** 1 Department of Pharmacology, Tulane University School of Medicine, New Orleans, LA 70112, USA; cgonza3@tulane.edu; 2 Lead Lab, Inc., New Orleans, LA 70119, USA; powellet2@gmail.com

**Keywords:** Cochrane Collaboration, Declaration of Helsinki, GIS, environmental signaling, exposome, lead intervention, urban mapping

## Abstract

This study appraises New Orleans soil lead and children’s lead exposure before and ten years after Hurricane Katrina flooded the city. *Introduction*: Early childhood exposure to lead is associated with lifelong and multiple health, learning, and behavioral disorders. Lead exposure is an important factor hindering the long-term resilience and sustainability of communities. Lead exposure disproportionately affects low socioeconomic status of communities. No safe lead exposure is known and the common intervention is not effective. An essential responsibility of health practitioners is to develop an effective primary intervention. *Methods*: Pre- and post-Hurricane soil lead and children’s blood lead data were matched by census tract communities. Soil lead and blood lead data were described, mapped, blood lead graphed as a function of soil lead, and Multi-Response Permutation Procedures statistics established disparities. *Results*: Simultaneous decreases occurred in soil lead accompanied by an especially large decline in children’s blood lead 10 years after Hurricane Katrina. Exposure disparities still exist between children living in the interior and outer areas of the city. *Conclusions*: At the scale of a city, this study demonstrates that decreasing soil lead effectively reduces children’s blood lead. Primary prevention of lead exposure can be accomplished by reducing soil lead in the urban environment.

## 1. Introduction

Lead is included in the dynamic inputs, storages, transformations, and outputs that form part of the interconnected processes by fashioning, via environmental signaling, the urban life-support environment, i.e., exposome [1,2]. This study appraises changes in New Orleans soil lead and children’s lead exposure before and ten years after Hurricane Katrina. Lead poisoning hinders the resilience and sustainability of communities because of its association with numerous life-long childhood-to-adulthood human health and behavioral disorders [3,4,5]. Lead disproportionately affects socioeconomic status of communities [4,6]. Furthermore, studies show strong associations between early childhood lead exposure and subsequent delinquency and criminal behavior in teenagers [7,8].

Understanding lead exposure has evolved, especially as it relates to children’s health. Between 1990 and 2012, the blood lead exposure guideline was 10 micrograms per deciliter (µg/dL). Then, in 2012, the Centers for Disease Control (CDC) confirmed that there is no known safe lead exposure and changed the “guideline value” to a “reference value”. The reference value is defined as the 97.5 percentile of the children’s blood lead from the National Health and Nutrition Examination Survey (NHANES). The current NHANES blood lead value is 5 µg/dL [9]. The CDC emphasizes the need for primary prevention and this requirement imposes substantial obligations on officials and the medical community to pursue multipronged approaches to prevent lead exposure.

Children’s exposure to lead occurs directly from soil by hand-to-mouth behavior. This behavior is an inherent characteristic of the developing nervous system [10]. Children are also lead exposed through dust inhalation, food, water, and other sources. Children require minerals such as calcium to support skeletal development and when calcium is in short supply, lead is absorbed in its place [11].

The common way to determine if intervention is required is by testing children’s blood lead. If blood lead is above the reference value, then steps proceed to inspect the home and arrange an intervention. Current intervention includes education and household lead dust control. There are two critical problems with this procedural method. First, using children’s blood to test for lead in the environment fails to meet the goal of primary prevention, which should require mitigation of the hazard in the first place, not merely demonstrate its presence in the environment after exposure. The second issue concerns the efficacy of the prescribed intervention. Studies on the effectiveness of medical interventions have been reviewed by the Cochrane Collaboration [12]. A thorough evaluation of the effectiveness of education and household dust cleaning data from fourteen studies plus another study indicates that the common program of lead intervention is not effective [12,13].

The current understanding about the childhood-to-adulthood lead hazards and the lack of effective intervention combine to challenge health agencies with issues about actions to reduce exposure. This study contributes to the knowledge about ecological factors affecting health disparities [14]. It builds on previous studies that evaluate dynamic changes of soil lead and blood lead in communities of New Orleans [15,16]. One research question concerns the relationship between soil lead and children’s blood lead in the context of disparities before and after Hurricane Katrina. Another research question concerns the application of experience from Hurricane Katrina for advancing intervention to prevent children’s exposure to soil lead and associated toxicants.

## 2. Materials and Methods

**Figure 1:**
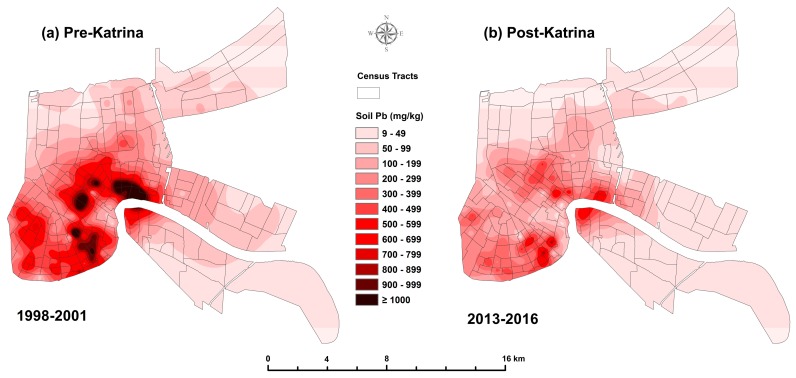
New Orleans soil lead exposome: (**a**) the pre-Katrina panel presents the kriged soil lead for the survey 1998–2000 (182 CTs). Note that some of the census tracts had median soil lead content ≥1000 mg/kg soil; (**b**) the post-Katrina panel presents the soil lead for the 2013–2015 survey (182 CTs). The reduction in soil lead changed the environment, i.e., exposome, of the city. Note the post-Katrina reduction of census tracts with exceptionally large median soil lead amounts (≥1000 mg/kg).

**Figure 2:**
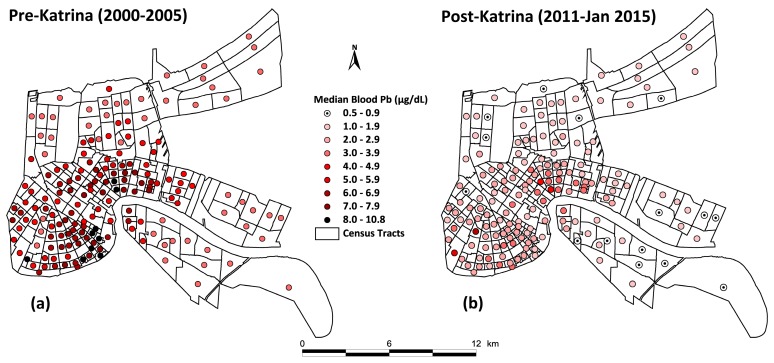
Children’s median blood lead by census tract pre- and ten years post-Katrina.: (**a**) the pre-Katrina (2000–2005) panel shows children’s blood lead by census tracts (n = 172) of New Orleans (164 CTs) and in St. Bernard (n = 8 CTs); (**b**) the post-Katrina (2011–January 2015) panel illustrates the median blood ten years after the flooding of 80% of the City of New Orleans. The census tracts shown are matched for soil lead and blood lead during the two time-periods.

**Figure 3:**
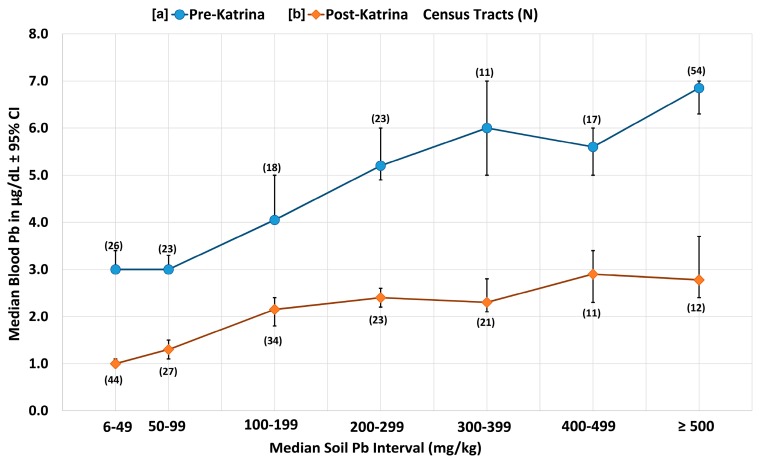
Soil lead and blood lead changes in Orleans Parish (164 CTs) and St. Bernard Parish (8 CTs) are illustrated for 172 census tracts matched for soil Pb and blood Pb data. The x-axis is median soil Pb intervals for 172 census tracts, matched with median blood lead intervals on the y-axis. There are two curves: (**a**) the top curve represents the matched pre-Katrina soil and blood lead data; and (**b**) the bottom curve summarizes the post-Katrina blood lead and soil lead results. The error bars are the 95% confidence intervals of the median. In parentheses, the number of census tracts within each interval of soil lead and blood are given. The differences in pre- and 10 years post-Katrina responses of children’s blood lead to soil lead is the result of multiple factors as discussed in Section 4.3.

**Figure 4:**
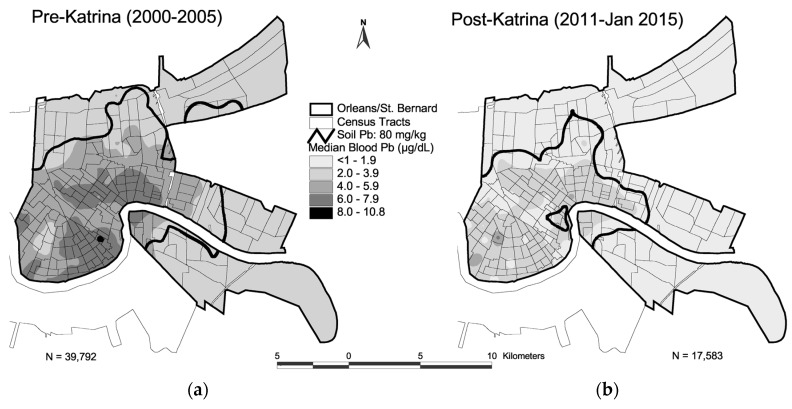
The 80 mg/kg isoline is based on the California soil lead guideline [20]. Panel (**a**) is for pre-Katrina (2000–2005) and shows the 80 mg/kg isoline and children’s median blood lead by census tracts (n = 172) in Orleans (164 CTs) and St. Bernard (n = 8 CTs); (**b**) the post-Katrina (2011–January 2015) panel illustrates the 80 mg/kg lead isoline and median blood ten years after 80% of New Orleans was flooded. Although there has been a consistently large decrease in soil lead and especially in children’s blood lead across all communities, lead exposure disparities continue within inner-city communities compared with outer communities of the city.

Address-geocoded blood lead data of unidentified children ≤6 years of age were provided by the Louisiana Healthy Homes and Childhood Lead Poisoning Prevention Program. This data was subsequently stratified by census tract [17,18]. Table 1 and Figure 1, Figure 2, Figure 3 and Figure 4 include soil lead and children’s blood lead data from New Orleans and adjacent St. Bernard Parishes (Parish = U.S. County).

Beginning in 1998, the research team collected soils samples at the rate of 19 per census tract from across New Orleans. By 2000 a soil lead map had been created for the area. A study described similarities between an earlier urban lead map completed in 1992 with the one completed in 2000 [19]. The 2000 map is the basis for the pre-Katrina data used in this study.

In August 2005, Hurricane Katrina flooded 80% of New Orleans, thereby creating a unique natural experimental condition not ethically possible by any scientific standard. Ten years after Katrina, the same research team was reinstated to remap New Orleans [15,16]. The current study compares matched data by census tracts (n = 172) for pre-Katrina and post-Katrina time periods. The data includes soil lead (n = 3238 and 3243, for pre- vs. post-Katrina, respectively), and blood lead (n = 38,861 and 17,473, for pre- vs. post-Katrina, respectively). The census tract percentiles for soil lead and blood lead data for pre- and post-Katrina were graphed to illustrate changes in the city. The State of California Human Health Screening Level for soil lead on residential properties is 80 mg/kg and used for interpolation of an 80 mg/kg isoline to illustrate both the pre- and post-Katrina changes in soil lead and associated blood lead in census tracts within the city [20]. Statistical analysis was by permutation procedures and Fisher’s Exact Tests [21].

## 3. Results

Table 1 lists the percentiles of the data for pre- and ten years post-Katrina (i.e., pre-K and post-K) for matched soil lead (SPb) and children’s blood lead (BPb) for Orleans Parish and St. Bernard Parish Louisiana (N = 172 census tracts or CTs). Comparing median pre- to post-Katrina, the SPb concentrations show a decrease from 289 to 143 mg/kg, and a median BPb decrease from 5 to 1.9 µg/dL Both decreases have extremely small Multi-Response Permutation Procedure for Blocked Data (MRBP) *p*-values (<10^−21^) [21]. Figure 1 and Figure 2 map soil lead and blood lead changes pre- and post-Katrina in New Orleans. The combined changes of SPb and BPb data are graphed in Figure 3. Figure 4 maps the 80 mg/kg SPb isoline and the median BPb of children by census tract in pre- and 10 years post- Hurricane Katrina.

### 3.1. Change in Soil Lead

Figure 1 is a map of soil lead before and 10 years after Katrina. The map includes Orleans (171 CTs) and St. Bernard Parishes (11 CTs). Soil lead is shown as a continuous surface. The median soil lead was placed at the centroid of their corresponding census tract and were interpolated by kriging with linear semivariogram [22].

### 3.2. Change in Children’s Blood Lead

Figure 2 illustrates changes in blood lead by census tract before and ten years after Hurricane Katrina of Orleans and St. Bernard Parishes, Louisiana (172 census tracts).

### 3.3. Graph of the Disparities of Pre- and Post-Katrina Soil Lead and Blood Lead in New Orleans

The association between children’s median blood lead and median soil lead is displayed in Figure 3. Of the total number of census tracts collected, there are 172 census tracts that have matching soil and blood lead data for pre- and post-Hurricane periods. Figure 3 shows that in pre-Katrina (1999–2001) there are 54 census tracts that had soil lead ≥500 mg/kg and 26 census tracts with a soil lead in the lowest 6–49 mg/kg category. In post-Katrina (2012–2015) there are 12 census tracts with soil lead ≥500 mg/kg and 44 census tracts in the lowest 6–49 mg/kg category. As shown in Table 1, the decrease of median soil took place from 289 mg/kg to 143 mg/kg (a factor of 2.02) while blood lead declined from 5.0 µg/dL to 1.9 µg/dL (a factor of 2.63) after Hurricane Katrina. As illustrated in Figure 3, the soil lead decrease by a factor of 2.02 and blood lead decrease by a factor of 2.63 is consistent and robust across all communities of Orleans and St. Bernard Parishes. The highest soil lead and blood lead census tracts represent the inner-city communities and the lowest soil lead and blood lead census tracts represent the outer city communities (see also Figure 1 and Figure 2).

The decreased children’s blood lead response by 2.63 to soil lead decrease by 2.02 after Hurricane Katrina is demonstrated by the differences in the curves shown in Figure 3. The processes that may have reduced children’s blood lead more markedly than soil lead are discussed in Section 4.3.

### 3.4. Map of Pre- and Post-Katrina Changes of Soil Lead and Blood Lead

Figure 4 maps the changes in the 80 mg/kg soil lead isoline within the city of New Orleans before and 10 years after Hurricane Katrina. In addition, the map includes the associated median children’s blood lead results throughout the city. Although there was a major decrease of soil lead and blood lead throughout the city, the lead exposure disparity between the interior and outlying communities of the city continues 10 years after Hurricane Katrina.

## 4. Discussion

### 4.1. National Health and Nutrition Evaluation Survey (NHANES) vs. New Orleans Blood Lead Trends

The CDC tracks national children’s blood lead trends by way of the National Health and Nutrition Evaluation Survey (NHANES). The NHANES trends were compared with the New Orleans trends from pre-Katrina, 1999–2002, through to post-Katrina, 2011–2014 [16]. The national trend of children’s blood lead underwent a decrease from a median blood lead of 2 µg/dL to 1 µg/dL. During the same years, the children of New Orleans underwent a much steeper decline of blood lead from 5.0 µg/dL to 1.7 µg/dL. The blood lead for the children of the nation declined by a factor of 2, while the blood lead for the children of New Orleans declined by a steeper factor of 2.9 [16]. The median blood lead for the children of New Orleans is around 0.7 µg/dL larger than the children of the nation. The New Orleans blood lead results are specifically associated with the geographic location of the population living in various urban communities, while the national blood lead results are associated with demographic characteristics of the nation’s children. The difference in data strategies raises questions about the outcome of blood lead response if instead the nation’s children were stratified geographically (i.e., urban, suburban, rural community) instead of by demographic characteristics of the population.

The lack of progress in reducing health disparities is a major concern. Population studies combined with exposure science approaches (various omics) and global modeling approaches are proposed to fill the gap in knowledge [23]. While the global modeling approach might provide insights into exposure factors related to lead and other diseases, methods are currently lacking that comprehensively identify correlations between exposure responses and the total environment, i.e., the exposome [23]. The effect of lead on health encompasses the life-long spectrum of human diseases [4,24]. Studying urban soil lead by communities and lead exposure of children within the same communities describes an underlying health disparity in the urban environment. Pre-Katrina research documented the association between soil lead and socioeconomic characteristics of people living in New Orleans [25]. Future research on the socio-economic changes 10 years after Hurricane Katrina would provide further insight into association between soil lead and social characteristics of communities.

### 4.2. Inputs of Lead and Other Toxic Substances into the Environment

The concept of the metabolism of cities was coined by Wolman, who applied it to urban waterworks [1]. The concept is broadened here to include the environmental signaling components of the industrial metabolism of cities which comprises the inputs, transformations, storages, and outputs of mainly anthropogenic toxic materials. Two major sources of lead contamination in residential communities are tetraethyl lead (TEL) additives (which contribute exhaust emissions of fine lead particles from vehicles that accumulated in soils) and lead-based paint (weathered or sanded, that is deposited in soil).

Tetraethyl lead is a particularly large input source of lead dust in cities. In 1925, a major decision was made to allow the use of TEL additives in gasoline. The proposal was vigorously protested by Yandell Henderson and other health scientists, but the scientific concerns of the protesters were dismissed [26]. The use of TEL began in 1925 and increased in proportion to the growth of vehicle use and the proliferation of highways.

Leaded paint and TEL are now regulated by federal legislation to limit their use. Lead-based interior paint is limited to 90 mg/kg nationally and TEL is banned for street and highway use, although it is still allowed in piston powered aircraft. Interior communities of all U.S. cities typically exhibit—in proportion to size—higher soil lead concentrations due to multiple lead sources, including five decades of lead dust accumulation from vehicle exhaust [27].

The pattern of soil lead in New Orleans is an outcome of all uses of lead. Comparison of the mass of TEL in vehicle fuels with the mass of lead in lead-based paint provides insight into the inputs, accumulation, and storage of lead in soils. In New Orleans at least 10 times more lead dust can be accounted for from TEL exhaust emissions (that were deposited into soil), than can be accounted for by lead dust potentially and continuingly from lead-based paints [28]. Lead dust contamination in New Orleans’ soil is directly associated with traffic flows; soil lead is largest in areas of the city with the highest rates of traffic congestion and smallest in outer areas of the city with historically low rates of traffic congestion. This urban pattern of lead has been described for cities throughout the nation [27,29].

The parent material of New Orleans soil is alluvial sediment from the Mississippi River. Research on modern alluvial sediments reveals that they contain small amounts (<10 mg/kg) of lead strongly correlated with other metals [30]. For example, the amount of soil zinc is closely correlated with soil lead [30]. Zinc inhibits plant growth; when lead and zinc coexist in soil, then reduced plant growth would be an expected outcome and the results would be a soil surface environment with more accessible soil lead. This means that although soil lead is a major component of the urban environment, it is strongly associated with other toxic substances such as polycyclic aromatic hydrocarbons that also exert health risks [31]. Thus, Figure 1 represents a combination of lead and other associated toxicants.

### 4.3. Soil Lead Content vs. Lead Loading, Environmental Signaling, and Lead Exposure

The larger post-Katrina decline of children’s blood lead (factor of 2.63) to soil lead (factor of 2.01) described in Section 3.3 may be the result of multiple circumstances including demographic changes of the population, decreases of lead dust in home interior environments after restoration/cleanup from flooding, and the possibility that small changes in soil lead transforms into larger reductions of lead dust re-suspension during droughty times of the year. To understand soil as a lead dust reservoir for environmental signaling, it is important to recognize that the common units of measurement thwart appreciation about the quantities of lead loading on the soil surface. Indoor dust samples are collected from the floor (i.e., surface loading) and the units are lead per area (i.e., µg/m^2^, or, in the U.S., µg/ft^2^). Soil samples are collected, analyzed, and reported as lead content, or amount of lead per mass; i.e., ppm, mg/kg, or µg/g. Research comparing soil lead content mg/kg (µg/g or ppm) with soil lead loading µg/area reveals the relationship between the two units of measurement [32]. The U.S. indoor lead dust standard is 40 μg/ft^2^ (~431 μg/m^2^) and recently revised downward to 10 μg/ft^2^ (~108 μg/m^2^). The current U.S. EPA standard for soil lead where children play is 400 mg/kg. For soil containing 400 mg/kg, the surface loading is ~16,200 μg/m^2^ (1508 µg/ft^2^) [32]. This means that the EPA soil lead standard poses a lead loading risk over 37 times larger (or 151 times larger for the revised floor standard) than allowed on interior floors of home environments. The differences in units of measurement expressed by lead content and lead loading creates a general misunderstanding about the size and potency of the soil reservoir of lead dust that acts as an environmental signal on children’s health. The topic of the relationship between soil lead content and lead dust loading may be a key for understanding how smaller incremental decreases of soil lead convert into larger incremental decreases of children’s blood lead before and after Katrina. The differences in blood lead responses to soil lead before and after Katrina, as shown in Figure 3, requires further research.

Children are responsive to soil lead for several reasons. First, they have an innate hand-to-mouth characteristic that is first expressed during fetal development [10]. Secondly, they are extremely sensitive to lead aerosols, as demonstrated by the fact that their blood lead responds directly to air lead and the inhalation route of exposure [33]. The lead content of soils—which varies from one community to another—drives the risk of hand-to-mouth and inhalation routes of exposure. The surface lead loading of soil has consequences related to the seasonal cycle of late summer increases of children’s blood lead. Lead contaminated soil resuspended to atmospheric lead has been demonstrated in several cities [33]. The processes involve seasonally dry weather in late summer, when the lead dust in surface soil is picked up by the wind and re-suspended into the air [34]. Seasonal increases in air lead results in inhalation of lead aerosols which transfers lead dust directly to the blood stream. In the Northern Hemisphere children’s blood lead is highest during July and August when soils are driest, and re-suspension most active, and lowest during winter months of January and February. An example of the seasonality phenomena is shown by the winter and summer changes in environmental signaling of lead dust and blood lead levels of children living in Detroit [35].

### 4.4. Lessons from Hurricane Katrina and the New Orleans Soil Environment

Revitalizing the urban environment involves confronting the damages caused by the millions of tons of lead deposited by multiple sources [27,31,36]. When children present with blood lead above desirable amounts, the common intervention employs a combination of education and household dust cleanup, but this intervention method has been deemed ineffective by Cochrane Collaboration review (see Appendix A). The *Declaration of Helsinki—Ethical Principles for Medical Research Involving Human Subjects* states that if an intervention is ineffective, then it is the obligation of the medical community to develop an alternative and effective intervention to replace the ineffective methods [37]. As demonstrated for New Orleans in Figure 3 and Figure 4, decreasing urban soil lead creates lead safer communities for children living and playing in the city [38]. Several methods are available for intervention of soil lead [39]. The lesson from Katrina is that the storm washed low lead soils from the coastal environment into the city and children’s blood lead underwent a reduction. Thus, transporting low lead soils into the city is an intervention action that can be undertaken. Several soil lead projects have been undertaken in New Orleans to improve children’s play areas [40,41]. As illustrated by Figure 1 and Figure 4, low lead soil is available in the outer areas of the city, and it is relatively inexpensive to transport it into the city. It is important to understand that in their outer and rural areas, all U.S. cities have low lead soil (<20 mg/kg) as demonstrated by soil metal studies conducted by the US Geological Service [42].

## 5. Conclusions

Soil lead in New Orleans declined sharply after Hurricane Katrina. The soil lead reductions were accompanied by simultaneous and larger decreases in children’s blood lead. This finding underscores the concept that soil is a major reservoir for lead dust and an exposure source to children. This information provides an intervention method; specifically, by reducing surface soil lead at the community scale to protect childhood populations from lead exposure, it is possible to diminish environmental health disparities, and to enact primary lead prevention.

## Figures and Tables

**Table 1:** Data for before and ten years after Hurricane Katrina (i.e., pre-Katrina and post-Katrina) for the matched soil lead (SPb) and children’s blood lead (BPb) data from Orleans Parish and St. Bernard Parish census tracts (CTs) in Louisiana. Eighty percent of New Orleans was flooded by the levee protection failures caused by Katrina’s storm surge.

Data Percentiles	Soil Lead (mg/kg) 172 CTs		Blood Lead (µg/dL) 172 CTs	
	Pre-Katrina	Post-Katrina	Pre-Katrina	Post-Katrina
min	11	10	3.0	<1.0
5%	26	16	3.0	<1.0
10%	41	24	3.0	1.0
25%	85	44	3.4	1.2
50%	289	143	5.0	1.9
75%	559	307	6.5	2.5
90%	806	419	7.4	3.0
95%	1052	593	8.1	3.4
max	1789	1076	10.8	6.8
N	3238	3243	38,861	17,544
*p*-value *		1.12 × 10^−21^		6.95 × 10^−37^

* Multi-Response Permutation Procedure for Blocked Data (MRBP) [21]. Probability of Pearson Type III error.

## Appendix A

Cochrane Collaboration Review Abstract from Reference 9, Education and household interventions for preventing domestic lead exposure in children [12].

**Why is this review important?**

Lead poisoning at high levels can cause anaemia, multi-organ damage, seizures, coma and death in children. At chronic low levels it can lead to cognitive, psychological and neurobehavioral impairment. Researchers have studied many different educational and environmental household interventions to prevent lead exposure in children, such as parental education, removal of lead dust or home remediation work. However, it is not clear if and to what extent these interventions work in preventing lead exposure in children.

**Who will be interested in this review?**

Parents and caregivers who want to prevent domestic lead exposure in children; health professionals and decision-makers who are interested in methods to prevent domestic lead exposure in children.

**What questions does this review aim to answer?**

We wanted to find out if educational or environmental household interventions, or combinations of both, are effective in preventing or reducing domestic lead exposure in children up to 18 years of age. We were interested in looking at improvements in cognitive and neurobehavioral development, reductions in blood lead levels and household lead dust levels.

**Which studies were included in the review?**

We searched databases up to May 2016 for randomized controlled trials, or RCTs (where participants are randomly assigned to treatment and control groups; in this case, with one group that does not receive any intervention and one or more other groups that do) and quasi-RCTs (where children are assigned to groups using methods that are not strictly random). We found 14 studies involving 2643 children, that investigated educational or environmental interventions, or a combination of both, to reduce domestic lead exposure in children. Children in all studies were under six years of age. Thirteen studies took place in urban areas of North America, and one was in Australia. Most studies were performed in areas with low socioeconomic status. Boys and girls were equally represented in all studies. The duration of the intervention ranged from 3 months to 24 months in 12 studies, and 2 studies performed an intervention on a single occasion. Follow-up periods ranged from 6 months to 48 months. National or international research grants or governments funded 12 studies, and 2 studies did not report their funding sources.

**What does the evidence from the review reveal?**

We did not find any studies that evaluated effects on cognitive or neurobehavioral outcomes or adverse events in children. All studies reported on blood lead levels. The included studies found that educational interventions are not effective in reducing blood lead levels of young children; the quality of this evidence was moderate to high. Dust control interventions may lead to little or no difference in blood lead levels; however, future research might change these results because the quality of evidence was moderate-to-low for these interventions. There is currently insufficient evidence that soil abatement or combination interventions reduce blood lead levels, and further studies need to address this.

**What should happen next?**

More research is needed to find out what is effective for preventing children’s exposure to lead. Studies should be carried out in different socioeconomic groups in high-, middle- and low-income countries to consider how interventions work in contexts shaped by different levels of industrialization or environmental and occupational health safety regulations.
